# Case Report: Successful Management of *Conidiobolus Lamprauges* Rhinitis in a Dog

**DOI:** 10.3389/fvets.2021.633695

**Published:** 2021-02-05

**Authors:** Jared A. Jaffey, Eric T. Hostnik, Aline Rodrigues Hoffman, Maureen Jay, Sylvia H. Ferguson, Nathan P. Wiederhold

**Affiliations:** ^1^Department of Specialty Medicine, Midwestern University College of Veterinary Medicine, Glendale, AZ, United States; ^2^Department of Veterinary Clinical Sciences, Veterinary Medical Center, Ohio State University, Columbus, OH, United States; ^3^Department of Veterinary Pathobiology, College of Veterinary Medicine & Biomedical Sciences, Texas A&M University, College Station, TX, United States; ^4^Department of Surgery, Animal Medical & Surgical Center, Scottsdale, AZ, United States; ^5^Department of Pathology and Population Medicine, Midwestern University College of Veterinary Medicine, Glendale, AZ, United States; ^6^Fungus Testing Laboratory, Department of Pathology and Laboratory Medicine, University of Texas Health Science Center, San Antonio, TX, United States

**Keywords:** conidiobolomycosis, fungal rhinitis, canine (dog), epistaxis, itraconazole, terbinafine

## Abstract

This is a case of *Conidiobolus lamprauges* rhinitis in a Goldendoodle, that was presented for evaluation of sneezing, coughing, lethargy, as well as right-sided epistaxis and clear ocular discharge. Computed tomography revealed a large amount of soft tissue within the right nasal passage that obscured the osseous turbinates from the right maxillary canine tooth to the right side of the choanae. Biopsies revealed eosinophilic granulomas with variable number of basophilic to negatively staining, septate, fungal hyphae with non-parallel walls and irregular branching that were subsequently determined to be *Conidiobolus lamprauges* via panfungal PCR and sequencing. Complete and sustained resolution of clinical disease was achieved after 75 days of systemic antifungal therapy. This report describes for the first time, important clinical features of a dog with nasal conidiobolomycosis that will facilitate its recognition, prognostication, and treatment in clinical practice.

## Background

Conidiobolomycosis is an uncommon infection in companion animals. The veterinary literature is limited to four single case reports in dogs with *Conidiobolus* spp. identified in one each of subcutaneous lesions, oral cavity, lungs, and multi-focally (trachea, lungs, small intestine, and maxilla) (1–4). *Conidiobolus* spp. are ubiquitous in soil and decaying plant material globally, but the highest occurrence is in the United Kingdom, India, and the East Coast of the United States (5). The principal site of infection in humans is the nasopharynx (5). This report aims to provide a thorough description of clinical, endoscopic, computed tomography (CT), and molecular diagnostic features in a dog with rhinitis caused by *Conidiobolus lamprauges*.

## Case Presentation

A 3-year-old, male-neutered, Goldendoodle was presented to the Animal Medical & Surgical Center with a 4-week history of sneezing, coughing, lethargy, as well as right-sided epistaxis and clear ocular discharge (day 1). Physical examination revealed a temperature of 100.8°F, heart rate of 100 beats/min, and a respiratory rate of 55 breaths/min. There was viscous blood-tinged discharge from the right naris with an ipsilateral marked reduction in air flow with no abnormalities identified with the left nasal passage. There was no evidence of nasal pain, asymmetry, or depigmentation of the nares. The remainder of the examination was unremarkable. The dog was housed indoors only and had lived in Arizona, California, and Colorado.

**Figure 1:**
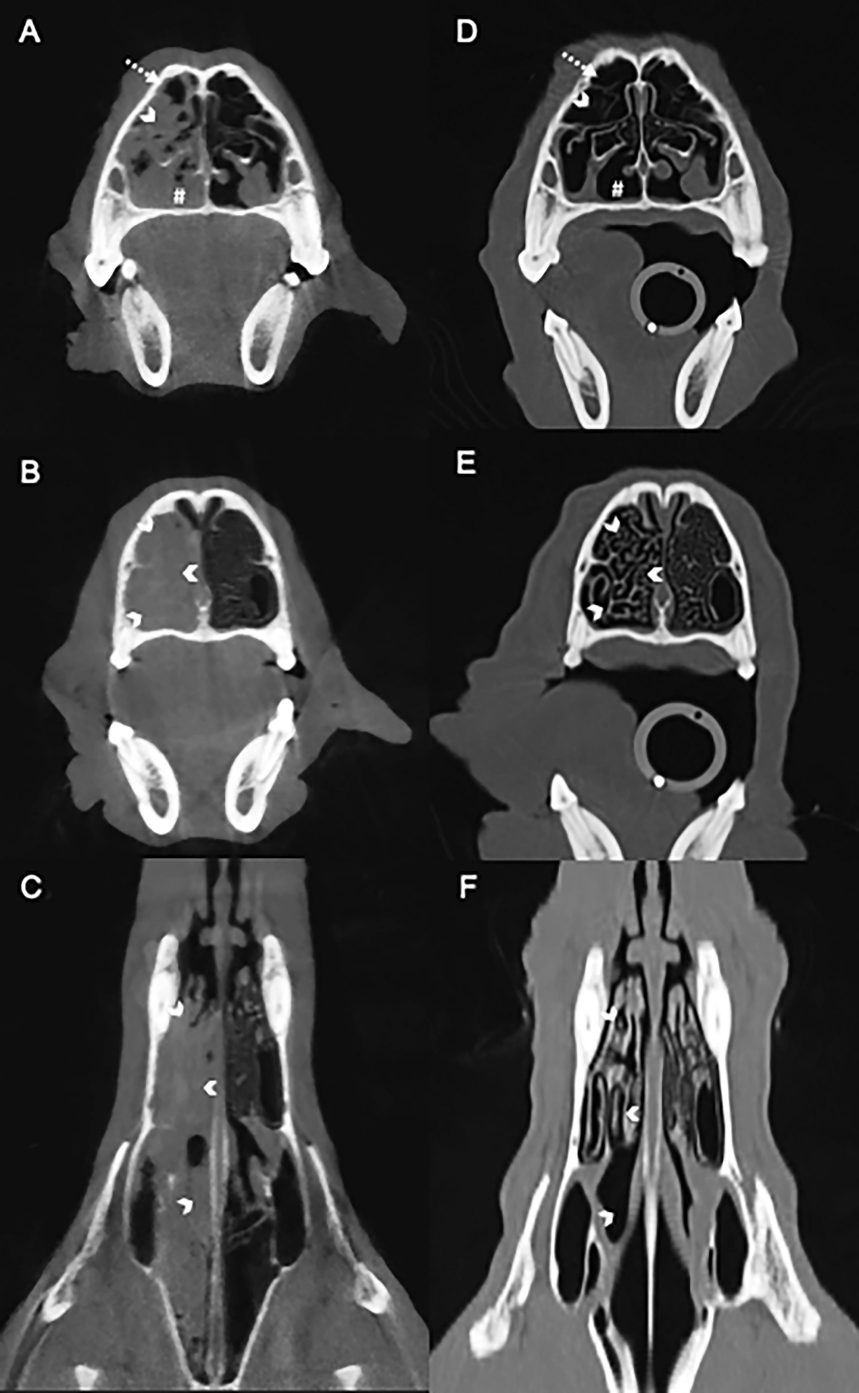
Computed tomography images of the nasal cavity of a dog with *Conidiobolus lamprauges* rhinitis. Selection of baseline images before antifungal therapy; no intravenous contrast medium was used in the initial evaluation **(A–C)**. Transverse **(A)** showing the unilateral nasal turbinates are obscured by the overlying soft tissue (white chevron) with small areas of gas in the non-dependent nasal passage (dashed arrow). The ventral nasal passage is obscured by soft tissue (white pound sign). Transverse **(B)** and dorsal **(C)** pretreatment showing the mucosa overlying the osseous nasal turbinates (white chevrons) eliminating gas between osseous scrolls. Selection of images after antifungal therapy **(D–F)**. Transverse post-treatment **(D)** highlighting persistent nasal turbinates (white chevrons) that were previously obscured in the pretreatment study **(A)**. Gas now fills the ventral nasal passage (white pound sign). Transverse **(E)** and dorsal **(F)** post-treatment showing the presence of gas between the nasal turbinates (white chevrons) with resolution of the soft tissue that had previously filled the nasal passages. Post-treatment images **(D–F)** demonstrate that the turbinates were obscured and that there is blunting of the osseous scrolls.

**Figure 2:**
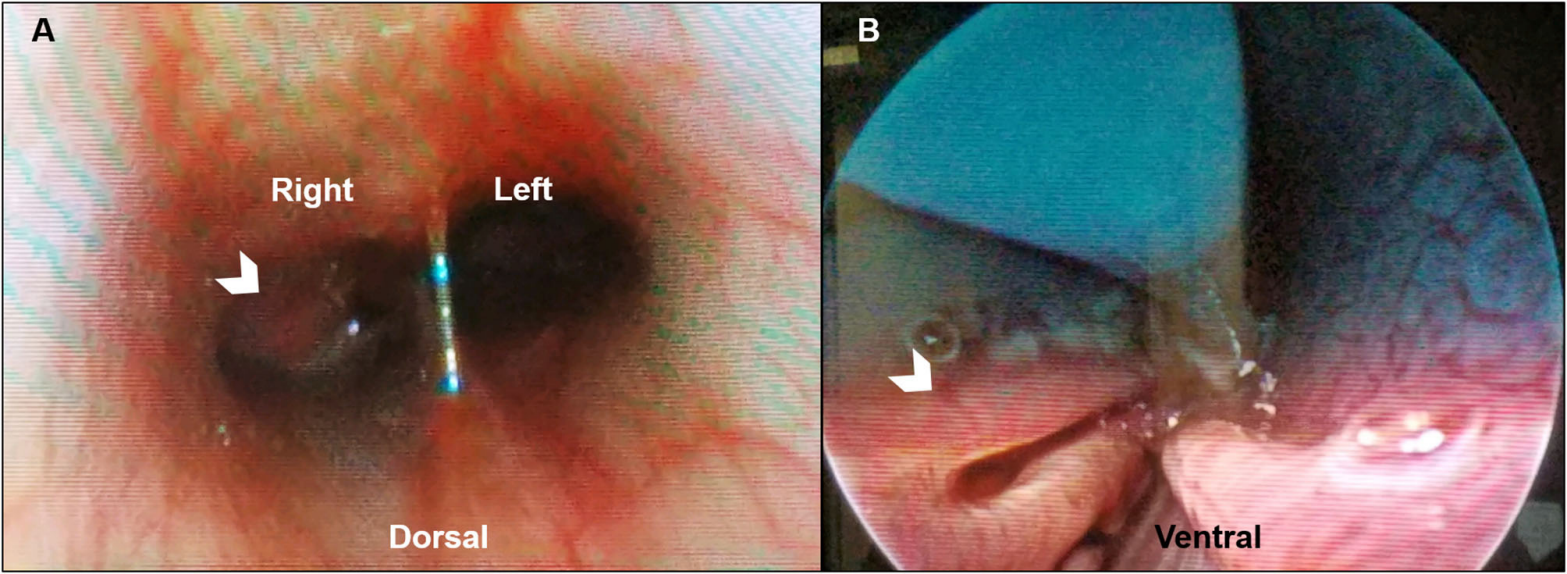
Representative posterior and anterior rhinoscopic images of a dog with *Conidiobolus lamprauges* rhinitis. **(A)** Posterior rhinoscopy with the dog in dorsal recumbency. Proliferative tissue extends caudally from the right side of the choanae (white chevron) with mild to moderately erythematous mucosa of the nasopharynx; **(B)** Anterior rhinoscopy in ventral recumbency. Turbinate mucosa that appears mildly erythematous and edematous (white chevron).

Clinically important complete blood count abnormalities were leukocytosis [18.6 ×10^3^/μL; reference interval (RI) 5.1–16.8 ×10^3^/μL], neutrophilia (12.7 ×10^3^/μL; RI 3.0–11.6 ×10^3^/μL), and eosinophilia (1.7 ×10^3^/μL; RI 0.1–1.2 ×10^3^/μL). Results from a serum biochemistry, urinalysis, coagulation testing (PT and PTT), and indirect systolic blood pressure were unremarkable. Computed tomography (CT scanner, NewTom 5G 2-slice, Cefka s.c., Imola, Italy) imaging of the skull was performed on day 1 and revealed a large amount of soft tissue within the right nasal passage that obscured the osseous turbinate scrolls from the right maxillary canine tooth root to the right side of the choanae (Figures 1A–C). The left nasal passage, septum, and cribiform plate were unremarkable (Figures 1A–C). The nasopharynx and choanae were then visualized with a flexible endoscope (Karl Storz Flexible Broncho-Fiberscope, Segundo, CA, USA). There was proliferative tissue that extended caudally from the right side of the choanae and the mucosa of the nasopharynx appeared edematous and erythematous (Figure 2). A small biopsy was retrieved from this region for histopathology. Next, antegrade visualization of the nasal passages was performed with rigid rhinoscopy (Karl Storz multi-purpose rigid scope 30) and the right nasal turbinates showed generalized marked erythematous and edematous mucosa with hemorrhage and mucopurulent material (Figure 2). No obvious fungal plaques or foreign bodies were visualized and the left nasal cavity appeared unremarkable. Blind biopsies were obtained from the right nasal passage for histopathology as well as aerobic/anaerobic and fungal culture with antimicrobial susceptibility. Differential diagnoses considered at that time included neoplasia, abscessation, atypical fungal rhinitis, or foreign body. The dog recovered uneventfully and was discharged with codeine (1.4 mg/kg PO q8–12 h ×5 days).

**Figure 3:**
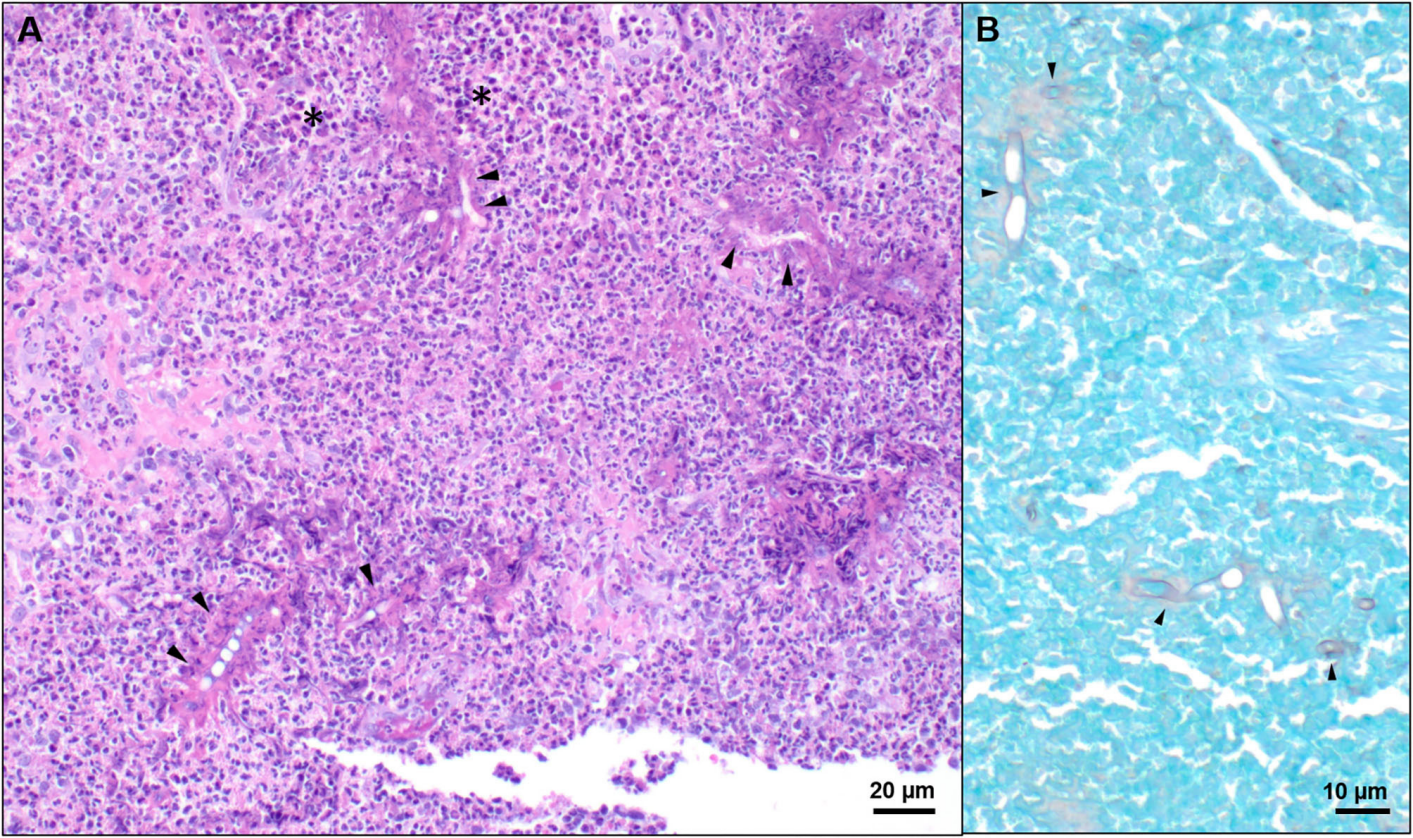
Representative histologic images of nasal tissue obtained from a dog with *Conidiobolus lamprauges* rhinitis. **(A)** Nasal tissue effaced by marked infiltrates composed predominately of eosinophils and epithelioid macrophages (black asterisks). Numerous 5–10 μm diameter, negatively staining to weakly basophilic hyphae were surrounded by a radiating “eosinophilic sleeve” (black arrows); hematoxylin and eosin stain. **(B)** Hyphae were irregularly septate, had non-parallel walls, irregular branching, and occasional ballooning dilations. GMS stain.

Histopathology (returned on day 8) revealed marked inflammatory infiltrates composed of large numbers of eosinophils, neutrophils, numerous epithelioid macrophages, multinucleated giant cells, lymphocytes, and plasma cells. Within these eosinophilic granulomas were variable number of basophilic to negatively staining, septate, fungal hyphae with non-parallel walls, irregular branching, and occasional ballooning dilations, ranging from 5–12 μm in diameter, often surrounded by a wide “eosinophilic sleeve” (Figure 3). A *Pythium insidiosum* ELISA was subsequently performed and was negative. Bacterial culture and antimicrobial susceptibility also returned at that time and revealed growth of methicillin-resistant *Staphylococcus pseudointermedius* on enrichment broth (Supplementary Table 1). The dog was initially prescribed fluconazole (6.1 mg/kg PO q12h) and marbofloxacin (3.1 mg/kg PO q24h ×14 days). However, on day 25, the dog was switched to itraconazole (9.2 mg/kg PO q24h), terbinafine (15.3 mg/kg PO q12h), and prednisone (0.6 mg/kg PO q24h ×7 days and then 0.3 mg/kg PO q24h).

The dog was presented to the Companion Animal Clinic at the Midwestern University College of Veterinary Medicine (on day 38 for evaluation of persistent clinical signs that included sneezing, coughing, and right-sided mucopurulent nasal discharge (epistaxis resolved). Physical examination revealed purulent crusts associated with the right naris and normal airflow bilaterally. The remainder of the examination was unremarkable. With the fungal culture negative (day 27), paraffin-embedded nasal tissue from the initial biopsies was submitted to the Dermatopathology Specialty Service at Texas A&M University for panfungal PCR and sequencing. Immunologic testing was pursued to screen the dog for a potential immunodeficiency. An immunoglobulin assay for IgA, IgM, and IgG was performed through the Animal Health Diagnostic Center of Cornell University and revealed immunoglobulins within their respective reference intervals. Medical management with itraconazole and terbinafine remained unchanged while the prednisone dosage was decreased to (0.15 mg/kg PO q24h) and tapered until it was discontinued 10 days later.

Panfungal PCR targeting the large subunit region of the fungal genome was performed on scrolls of formalin-fixed, paraffin-embedded tissue, as described previously (6). The PCR product was submitted for sequencing and a 600 base pairs of the resulting sequence contig was analyzed with the Basic Local Alignment Search Tool using the National Center for Biotechnology Information database. The sequence matched *Conidiobolus lamprauges* with 99% base-pair match to CBS 728.97 and CBS 461.97 [GenBank Accession Nos. MH874275.1 (597/602 base-pair match) and MH874268.1 (595/604 base-pair match), respectively]. The sequence was deposited in the GenBank database under accession number MT471266.

On day 61, the dog was presented for a planned evaluation and the owner reported the dog had improvement in severity and frequency of sneezing, coughing, and nasal discharge. Mild crusting of the right naris was the only abnormality on physical examination. A CT (CT scanner, Syngo VC 40 16-slice, Siemens Healthcare, Germany) of the skull, thorax, and abdomen as well as rhinoscopy were performed to assess the extent of residual disease in the nasal cavity and to search for evidence suggestive of dissemination.

The soft tissue of the right nasal passage had resolved and the right nasal turbinates were mildly blunted with slight widening of the spacing between the osseous scrolls (Figures 1D–F). The thoracic and abdominal images were unremarkable. Following the CT, the choanae and nasopharynx were visualized with a flexible endoscope (Karl Storz Flexible Silver Scope Small Flexible Gastrointestinal scope, Segundo, CA, USA) and appeared unremarkable. Antegrade visualization of the nasal cavities was achieved with a rigid endoscope (Karl Storz multi-purpose rigid scope 30) and revealed mild mucosal edema and erythema as well as minimal turbinate atrophy and bridging. A repeat biopsy of the nasal mucosa was obtained for routine histopathology and the sections were microscopically unremarkable. Grocott's methenamine silver (GMS) staining was also performed and fungal hyphae were not observed within any of the examined sections. The persistence in clinical signs and mild rhinoscopic abnormalities prompted the recommendation to continue terbinafine and itraconazole at unchanged dosages.

The last in-hospital recheck examination took place on day 83. The owner reported no clinical abnormalities and the physical examination was unremarkable. Treatment with antifungal medications remained unchanged and a recheck examination was requested for 2–3 months; however, the owner discontinued all medications without veterinary consultation on day 116. Follow-up with the owner by phone at the time of this writing (day 347) revealed that the dog had no evidence of clinical disease.

## Discussion

This report documents the clinical, endoscopic, CT, and molecular diagnostic features of *Conidiobolus lamprauges* rhinitis in a dog. *Conidiobolus* spp. are members of the Order Entomophthorales (7). The three main species that have been reported to lead to clinical conidiobolomycosis in humans and animals are *Conidiobolus coronatus, Conidiobolus incongruous*, and *Conidiobolus lamprauges* (2, 7, 8). This fungus is both invasive and saprobic, being found in soil, decayed plants, feces from amphibians, and even parasitically in insects (5, 9). The primary mode of transmission is believed to be inhalation of spores and possibly through wounds from mild trauma (5). This was evident in the case reported here in which disease was limited to the nasal cavity as well as in two other reports in dogs that identified *Conidiobolus* sp. in the trachea, lungs, or both (2, 3).

Fungal culture of infected tissue is the gold standard criterion to diagnose *Conidiobolus* sp.; however, hyphae from this genus are easily susceptible to damage from tissue homogenization (5). This is one possible explanation for the absence of growth on the fungal culture performed on the dog in this report. The histologic morphology of the hyphae in this case was suggestive of either an oomycete infection (e.g., *Pythium insidiosum*) (10) or those caused by Entomophthorales. It has been previously described that zygomycetes are more likely to have a wide “eosinophilic sleeve” around the hyphae (11). Furthermore, the presence of occasional ballooning hyphal dilations, as was observed in this case, is considered more characteristic of *Conidiobolus* spp. (12). The ability to perform panfungal PCR on formalin paraffin embedded tissue allowed for identification of the cause of the fungal infection in this case (6). It is important to identify the specific fungal organism in these cases, as oomycosis often fail to properly respond to antifungal treatments, whereas Enterophtomorales are more likely to respond. When organisms are seen histologically, but fungal culture fails, these alternative methods should be used to confirm the diagnosis, as it could impact treatment and prognosis.

The soft tissue of the right nasal passage created a mass effect in this dog, which made it difficult to differentiate between thickened nasal mucosal lining and a soft tissue structure such as a granuloma. Intravenous contrast medium was not administered at the time of the initial CT study, which could have helped to differentiate mucosal enhancement and osseous scrolls from luminal soft tissue. The soft tissue contouring to the osseous scrolls with thin mineral attenuations was suggestive of mucosal thickening causing the mass effect in this case. In limited human case reviews with nasal imaging available, *Conidiobolus* spp. infections resulted in either nasal mucosal thickening or granuloma formation without aggressive lysis of underlying bone (13–15). Findings in this dog were similar, the predominant change was the increased soft tissue within the affected nasal passageway lacking the lysis of the associated bony structures. The CT findings differed from a more commonly diagnosed fungal organism like *Aspergillus fumigatus*. Computed tomography findings for sinonasal aspergillosis in dogs commonly result in moderate to severe cavitary destruction of the turbinates with abnormal soft tissue in the nasal passages, thickening of the mucosa along interior surfaces of the frontal sinus, maxillary recesses and nasal cavity as well as thickened reactive bone (16). Comprehensive studies evaluating CT features of other atypical nasal fungal infections are lacking in the veterinary literature.

*Conidiobolus lamprauges* is primarily a pathogen of sheep and horses, but this species has also been reported to cause invasive disease in immunocompromised humans (17–19). In immunocompetent hosts, *Conidiobolus* spp. cause rhinofacial disease (5, 20). However, in the setting of immunosuppression, invasive disease similar to that caused by members of the order Mucorales (i.e., mucormycosis) can occur (21–23). *Conidiobolus* spp. have been shown to produce numerous putative virulence factors *in vitro*, lipolytic and proteolytic enzymes and are also thermophilic at 37°C (20, 24). The route of infection is *via* inhalation of spores or from minor trauma. Rhinoentophthoramycosis usually begins with a painless swelling that can progress over time with firm subcutaneous nodules in nasal and paranasal structures (5). In a meta-analysis that included 199 human cases of localized and systemic conidiobolomycosis, Choon et al. reported that 83% of cases involved otherwise healthy adults with nasal symptoms and central facial swellings (25). Improvement or cure was reported in most, after antifungal treatment, surgical resection, or both. In horses and sheep, infections have been reported to be localized to the nasopharyngeal area and are associated with the Splendore-Hoeppli phenomenon, an eosinophilic reaction around the invading hyphae and a characteristic histopathologic feature of the chronic subcutaneous lesions (5, 20).

Successful medical intervention in humans with rhinofacial conidiobolomycosis have included the use of one or more of the following: itraconazole (13, 14, 26–30), fluconazole (14), terbinafine (27, 30), potassium iodide (27–29), and amphotericin B (29). There are limited reports in the veterinary literature that describe medical treatments for conidiobolomycosis. Pneumonia caused by *Conidiobolus* sp. in a dog was successfully treated with itraconazole (3) and treatments in horses have included either fluconazole or intralesional injection with amphotericin B ± potassium iodide (31–33). The dog in this report was initially treated with fluconazole for 17 days before a switch to itraconazole and terbinafine. The change in therapy was motivated by concern that fluconazole would be ineffective provided the lack of clinical progress. The dog in this case was switched to a combination protocol that included itraconazole and terbinafine because of recommendations that originate from anecdotal reports of success with its use in dogs with other opportunistic fungal infections (34, 35). Terbinafine could act synergistically with azoles by amplifying ergosterol inhibition via different mechanisms but also provide fungicidal activity with an accumulation of squalene (34). Although there have been case reports of successful treatment with azoles, including fluconazole, the azoles have limited to no *in vitro* activity against *Conidiobolus* spp. (36), and some cases have also involved debridement and surgical resection (12). Interestingly, terbinafine alone has been reported to have modest *in vitro* activity against *Conidiobolus* spp. (37), and this agent has been used in combination with other antifungals (amphotericin B and itraconazole) in the treatment of rhinofacial conidiobolomycosis in humans with success (30, 38).

The dog in this report demonstrated complete resolution of clinical disease after 75 days of systemic antifungal therapy and remained apparently healthy 231 days after discontinuation of therapy. The mean treatment length until complete resolution of clinical disease in humans with rhinofacial conidiobolomycosis is 6.3 months (range, 0.8–18.0 months)(11–14, 24–30). In a previously reported dog with a *Conidiobolus* sp. infection, pneumonia had a rapid and complete response to itraconazole, although specific details regarding the length of antifungal treatment was not reported (3). The use of fluconazole to treat two mares with nasal conidiobolomycosis yielded complete and sustained resolution of disease after 6 and 8 weeks, respectively (31). The case reported here and other cases of nasal conidiobolomycosis suggest that prognosis with this disease is favorable in immunocompetent hosts.

## Conclusion

In conclusion, this report highlights the clinical, endoscopic, CT, and molecular diagnostic features of *Conidiobolus lamprauges* rhinitis in a dog. These results will facilitate recognition, prognostication, and treatment of this atypical fungal rhinitis in clinical practice.

## Data Availability Statement

The sequence was deposited in the GenBank database under accession number MT471266. Otherwise, all datasets generated for this study are included in the article/Supplementary Material.

## Author Contributions

JJ, EH, AH, MJ, SF, and NW: medical diagnosis, writing and editing manuscript, and review of final submission. All authors contributed to the article and approved the submitted version.

## Conflict of Interest

The authors declare that the research was conducted in the absence of any commercial or financial relationships that could be construed as a potential conflict of interest.
