# Supplementary material for: Case Report: Successful Management of Conidiobolus Lamprauges Rhinitis in a Dog
Source: Front Vet Sci. 2021 Feb 5;8:633695. doi: 10.3389/fvets.2021.633695 (PMC7892434; doi:10.3389/fvets.2021.633695)
Supplement: Supplementary file 1 [file Table_1.DOCX]

**Supplemental Table 1.** Aerobic and anaerobic bacterial culture and sensitivity results obtained from nasal tissue of a dog diagnosed with *Conidiobolus lamprauges* rhinitis.

|  | **Isolate organism** |  |  |
| --- | --- | --- | --- |
|  | Methicillin-resistant *Staphylococcus pseudointermedius* |  |  |
| **Antimicrobic** | **Results** | **Lowest value** | **Highest value** |
| Amikacin | ≤ 4 (S) | 16 | 32 |
| Ampicillin | > 0.5 (R) | 0.25 | 0.50 |
| Amoxicillin | > 0.5 (R) | 0.25 | 0.50 |
| Cefadroxil | --- | --- | --- |
| Cefazolin | --- | --- | --- |
| Cefovecin | --- | --- | --- |
| Cefpodoxime | --- | --- | --- |
| Cephalexin | --- | --- | --- |
| Chloramphenicol | ≥ 32 | 8 | 32 |
| Amoxicillin/Clavulanic Acid | --- | --- | --- |
| Clindamycin | ≥ 4 (R) | 0.5 | 2 |
| Doxycycline | ≥ 0.5 (R) | 0.12 | 0.5 |
| Enrofloxacin | 1.0 (I) | 0.5 | 2.0 |
| Gentamicin | ≥ 8.0 (R) | 2 | 8 |
| Marbofloxacin | ≤ 1.0 (S) | 1 | 4 |
| Mupirocin | ≥ 8.0 (R) | 4 | 8 |
| Potentiated sulfonamide | ≥ 1.0 (R) | 0.5 | 2 |

S, susceptible; I, intermediate; R, resistant
